# miR-27b-3p reduces muscle fibrosis during chronic skeletal muscle injury by targeting TGF-βR1/Smad pathway

**DOI:** 10.1186/s13018-024-04733-9

**Published:** 2024-06-02

**Authors:** Hang Yao, Jin Qian, Xu-ting Bian, Lin Guo, Kang-lai Tang, Xu Tao

**Affiliations:** Center of sports, Southwest Hospital, Army Medical University, Gaotanyan Str. 30, Chongqing city, 400038 People’s Republic of China

**Keywords:** MicroRNAs, Fibro-adipogenic progenitors, Fibrosis, Muscle injury

## Abstract

**Background:**

Fibrosis is a significant pathological feature of chronic skeletal muscle injury, profoundly affecting muscle regeneration. Fibro-adipogenic progenitors (FAPs) have the ability to differentiate into myofibroblasts, acting as a primary source of extracellular matrix (ECM). the process by which FAPs differentiate into myofibroblasts during chronic skeletal muscle injury remains inadequately explored.

**Method:**

mouse model with sciatic nerve denervated was constructed and miRNA expression profiles between the mouse model and uninjured mouse were analyzed. qRT/PCR and immunofluorescence elucidated the effect of miR-27b-3p on fibrosis in vivo and in vitro. Dual-luciferase reporter identified the target gene of miR-27b-3p, and finally knocked down or overexpressed the target gene and phosphorylation inhibition of Smad verified the influence of downstream molecules on the abundance of miR-27b-3p and fibrogenic differentiation of FAPs.

**Result:**

FAPs derived from a mouse model with sciatic nerves denervated exhibited a progressively worsening fibrotic phenotype over time. Introducing agomiR-27b-3p effectively suppressed fibrosis both in vitro and in vivo. MiR-27b-3p targeted Transforming Growth Factor Beta Receptor 1 (TGF-βR1) and the abundance of miR-27b-3p was negatively regulated by TGF-βR1/Smad.

**Conclusion:**

miR-27b-3p targeting the TGF-βR1/Smad pathway is a novel mechanism for regulating fibrogenic differentiation of FAPs. Increasing abundance of miR-27b-3p, suppressing expression of TGF-βR1 and inhibiting phosphorylation of smad3 presented potential strategies for treating fibrosis in chronic skeletal muscle injury.

**Supplementary Information:**

The online version contains supplementary material available at 10.1186/s13018-024-04733-9.

## Introduction

Skeletal muscle comprising over 600 pieces and accounting for 35-45% of body mass is the largest organ in the human body. It is composed of muscle fibers, muscle-resident cells, extracellular matrix (ECM), blood vessels, nerves, and other extracellular components, playing a crucial role in movement, respiration, energy metabolism and temperature regulation [1; 2]. Maintaining the homeostasis of skeletal muscle, that is its anatomical and functional integrity, is essential for sustaining life. However, various factors, such as local damage to cells and structures, as well as systemic signals from other organs or tissues, can easily influence skeletal muscle homeostasis [3–5].

Muscle stem cells, also known as satellite cells, can rapidly transition from a quiescent state to proliferative myogenic cells upon injury. However, the repair and successful regeneration of muscle fibers require collaboration with other resident muscle cells, including fibro-adipogenic progenitors (FAPs), fibroblasts, macrophages, and endothelial cells as demonstrated in models of acute muscle injury induced by cardiotoxin (CTX) or contusion [6–11]. The activated resident cells of the acute phase must be cleared to restore the original microenvironment of skeletal muscle after fiber regeneration [12]. Recurrent damage and inadequate treatment of underlying conditions can lead to the progression of acute injury to chronic injury, resulting in delayed clearance of resident cells, disrupted inter-cellular coordination, and ultimately pathological repair [13].

Fibrosis, characterized by abnormal deposition of ECM, is a significant pathological repair of chronic muscle injury. It hinders muscle regeneration, leading to muscle atrophy and weakness, ultimately impacting patients’ mobility [14]. Among the resident cells in muscle tissue, FAPs have emerged as key players in normal muscle repair. They not only differentiate into myofibroblasts and contribute to ECM production but also enhance the regenerative potential of satellite cells [15, 16]. Therefore, understanding how to effectively suppress the fibrogenic differentiation of FAPs, rather than just clearing them, is crucial in the context of chronic muscle injury. However, current research on the regulatory mechanisms governing FAPs’ differentiation into myofibroblasts is limited, highlighting the need for further exploration in this area.

MicroRNAs (miRNAs) are a class of endogenous non-coding RNAs approximately 22 nucleotides long. They are involved in various physiological processes by binding to or degrading the mRNA of target genes through the 3’ untranslated region (3’-UTR) [17, 18]. There is growing evidence demonstrating the significant role of miRNAs in organ fibrosis, including the lungs, kidneys, and heart [19–21]. For example, overexpression of miR-27b-3p alleviated the unilateral ureteric obstruction-induced mice renal fibrosis by suppressing transducers and activators of transcription 1 (STAT1) [22]. And miR-27b-3p also plays an equally important role in fibrosis of heart and lung [23, 24]. Studies have indicated that reduced expression of miR-29 in patients with Duchenne muscular dystrophy (DMD) and mdx mice suggested a potential role for miR-29 in skeletal muscle fibrosis [25]. Researchers have successfully delivered miR-146a-5p to mice with renal fibrosis using nanoparticles, leading to a significant reduction in renal fibrosis area and expression of actin alpha 2 (acta2) [26]. Additionally, miR-22-3p targeted Kruppel-like factor 6 (KLF6) to inhibit adipogenic differentiation of FAPs by downregulating Matrix metalloproteinase 14(MMP14) expression [27]. Moreover, dysregulation of miR-21-5p, miR-20a-5p, and miR-199a-5p during spinal cord injury can result in osteogenic differentiation of FAPs [28]. Therefore, it is reasonable to hypothesize that miRNAs may influence the fibrogenic differentiation of FAPs upon chronic skeletal muscle injury.

Here we discovered a progressive decrease of abundance of miR-27b-3p in FAPs extracted from the tibialis anterior (TA) of mouse model with sciatic nerve denervated, and fibrosis was significantly reduced both in vivo and vitro when transfected with agomiR-27b-3p. Furthermore, we found that miR-27b-3p targeted the transforming growth factor β receptor 1 (TGF-βR1) and regulated differentiation of FAPs into myofibroblasts through the TGF-βR1/Smad pathway. This finding has potential implications in the clinical treatment of skeletal muscle fibrosis and the translation of miRNA-based therapeutics.

## Materials and methods

### Animal experiment

All animal experiments and procedures were conducted in accordance with the guidelines and regulations set forth by the Institutional Animal Care and Use Committee of Army Medical University. 6 to 8-week-old C57BL/6 mice were utilized, and they were housed in a facility free from pathogens. To induce mouse model with sciatic nerve denervated, the following procedure was followed: mice were anesthetized with 0.5% (w/v) pentobarbital sodium. A 3 mm incision was made between the ischial tuberosity and the greater trochanter to expose the sciatic nerve located on the deep side of the gluteus maximus. A 5 mm segment of the nerve was excised before any branching occurred. This procedure was repeated on the contralateral side. For the interference experiment involving agomiR-27b-3p and agomiR negative control (agomiR NC) (Cat. No. B06004, GenePharma), they were diluted in DEPC water to the final concentration specified by the manufacturer’s protocol. Following nerve denervation, agomiR-27b-3p and agomiR NC were injected into the tibialis anterior (TA) muscle at a volume of 50 µl per muscle twice a week. TA samples were collected at 1 week, 2 weeks, 3 weeks, and 4 weeks post-denervation, denoted as DEN-1 W, DEN-2 W, DEN-3 W, and DEN-4 W, respectively, for further analysis.

### Cell isolation and FACS

FAPs were isolated following a previously documented procedure [29]. Hind limb muscles were obtained from uninjured C57BL/6 mice aged 6–8 weeks, and nerves and fat tissues were carefully excised. The muscles were then cut into approximately 1 mm^3-sized fragments. Collagenase II (Cat. No. C7806, Sigma) was employed to enzymatically digest the minced tissue, which was agitated at 500 rpm using magnetic stirring at 37 °C for 1 h. The resulting muscle suspension was filtered through a 100 μm and 40 μm cell strainer (Cat. No. 431,752 and 431,750, BD Bioscience) to remove any debris. Red blood cell lysis buffer (Cat. No. C3702, BD Bioscience) was utilized to eliminate red blood cells, and the cells were then resuspended in PBS. The cells were incubated with fluorescently-labeled antibodies in the dark at 4 °C for 30 min. The antibodies used included Alexa Fluor 488-CD31 (Cat. No. 160,208, Biolegend), Alexa Fluor 488-CD45 (Cat. No. 160,306, Biolegend), APC-Integrinα7 (Cat. No. FAB3518A, R&D), and APC-Cy7-Sca-1 (Cat. No. 108,126, Biolegend). The stained cells were analyzed using FACSAria III (BD Biosciences, NJ, USA), and the gating strategy was based on CD31-CD45-Integrinα7-Sca-1+ (supplementary Fig.  1a). FAPs from DEN-1 W, DEN-2 W, DEN-3 W, and DEN-4 W were isolated and analyzed following the established protocol.

### Cell culture and transfection

Freshly sorted FAPs from uninjured mice and 293T cells specifically utilized for dual luciferase assays were cultured in Dulbecco’s Modified Eagle Medium supplemented with 20% Fetal Bovine Serum (Cat. No. SH30406.05, HyClone) and 1% penicillin-streptomycin (Cat. No. C0222, Biolegend). The experimental timelines commence from the initiation of fresh FAP culture, designated as D0, with subsequent days labeled accordingly (e.g., D2, D3, and so forth). (1) Transfection with agomir-27b-3p or agomiR NC (Cat. No. B06004, GenePharma): On D2, the culture medium of freshly sorted FAPs was refreshed, and recombinant TGF-β protein (Cat. No. 594,509, Biolegend) at a final concentration of 5ng/ml in PBS was introduced to induce differentiation of FAPs into myofibroblasts. On D3, transfection reagent (Cat. No. AD600150, ZETA) was employed to transfect agomir-27b-3p or agomiR NC following the manufacturer’s guidelines. (2) Transfection with small interfering RNA targeting TGF-βR1 (si TGF-βR1) or scRNA custom-made from Sangon Biotech: The transfection procedure and timeline for si TGF-βR1 or scRNA mirrored those of the transfection with agomir-27b-3p or agomiR NC. (3) Transfection with plasmid of high expression TGF-βR1 or empty vector (Cat. No. C05007, GenePharma) in combination with agomir-27b-3p or agomiR NC: On D2, the culture medium of fresh FAPs was refreshed, and the TGF-βR1 plasmid or empty vector was transfected following the manufacturer’s protocol. On D4, agomir-27b-3p or agomiR NC was transfected to establish four distinct experimental groups: TGF-βR1 plasmid (+) agomir-27b-3p (+), TGF-βR1 plasmid (+) agomiR NC (+), vector (+) agomir-27b-3p (+), and vector (+) agomiR NC (+). The transfection reagent utilized was the same as before. (4) Utilization of a smad3 phosphorylation inhibitor: SIS3 (Cat. No. CAS 521984-48-5, targetmol), an inhibitor of smad3 phosphorylation formulated in DMSO, was introduced to freshly sorted FAPs at a final concentration of 4 μm on D2. On D3, the cells were transfected with agomir-27b-3p or agomiR NC as previously described.

### Enzyme-linked immunosorbent assay

To quantify the TGF-β concentration in denervated muscles, the TA was harvested and weighed at DEN-1 W, DEN-2 W, and DEN-4 W. Subsequently, the muscle samples were placed in separate Eppendorf (EP) tubes, and 300 µl of sterile PBS was added. The samples underwent sonication on ice using an ultrasonic processor (Q500, QSONICA) for 10 pulses at a power of 40 watts for 3 s each, with a 10-second interval between pulses. Following sonication, the tissue homogenate was centrifuged to collect the supernatant. The concentration of TGF-β1 was determined using an enzyme-linked immunosorbent assay (ELISA) kit for TGF-β1 (Cat. No. DB100C, R&D Systems) according to the provided instructions. The absorbance of the samples was measured three times within 30 min after adding the Stop Solution using a plate reader set to 450 nm. The TGF-β concentration was calculated based on the standard curve generated from the assay and then normalized to the muscle mass (mg).

### CCK-8 assay

In the Cell Counting Kit-8 (CCK-8) assay, 1 × 10^4 freshly isolated FAPs from uninjured mice were cultured and transfected with either agomir-27b-3p or agomiR NC following the previously described protocol in a 96-well plate. Subsequently, 10 µL of CCK-8 solution (Cat. No. HY-K0301-5mL, MedChemExpress) was added to each well. The wells containing complete medium and CCK-8 solution served as blank controls. The cells were then incubated in the dark at 37 °C with 5% CO_2_ for 2 h. After the incubation period, the optical density (OD) values at 450 nm were measured using a multi-function plate reader (Varioskan Flash, Thermo Fisher Scientific, USA). Given that the maximum transfection time with agomir-27b-3p or agomiR NC in FAPs is 72 h, the OD values were assessed at 0 h, 24 h, 48 h, and 72 h.

### Dual-luciferase reporter assay

The putative binding site of miR-27b-3p on the wild-type (WT) PDGFRα 3’UTR sequence and TGF-βR1 3’UTR, along with their corresponding mutated sequences (UGACACU), were individually cloned into the pMIR-REPORT Luciferase vector provided by OBIO Scientific Services. Subsequently, 293T cells were co-transfected with either Pmir-REPORT Luciferase-TGF-βR1 3’UTR (WT) or pMIR-REPORT Luciferase-TGF-βR1 3’UTR (MUT), along with agomiR-27b-3p or agomiR NC, for a duration of 48 h following the manufacturer’s protocol. Luciferase activities were quantified using a microplate reader. The dual-luciferase reporter assay conducted for PDGFRα mirrored the experimental setup for TGF-βR1.

### Immunohistochemistry, immunocytofluorescence and imaging

For immunohistochemical examination, fresh-frozen muscle tissues were sectioned into 8 μm slices using a cryostat (CM3050S, Leica, Germany). These tissue sections were fixed in 4% paraformaldehyde (PFA) for 5 min, permeabilized in 0.5% Triton X-100 (Cat. No. CS0913, BIOSIC) in PBS for 10 min, and subsequently blocked in a solution of PBS containing 10% normal donkey serum, 3% bovine serum albumin (BSA), and 0.1% Triton X-100 for 1 h at 37 °C. Following this, the sections were incubated overnight at 4 °C with the primary antibody, then washed with PBS and exposed to the secondary antibody tagged with Alexa Fluor™ 555 or 488 for visualization. Post-secondary antibody incubation, the sections underwent further PBS washes and were counterstained with Hoechst33342 (Cat. No. C1026, Beyotime) for nuclear visualization. In the case of immunocytofluorescent analysis, cells cultured on coverslips within a petri dish were rinsed with PBS, fixed in 4% paraformaldehyde (Cat. No. P0099, Beyotime) for 20 min, and processed following a similar protocol as the immunohistochemical analysis. The primary and secondary antibodies utilized were col1 (Cat. No. AF7001, Affinity), donkey polyclonal anti-rabbit IgG linked to Alexa Fluor™ 555 (Cat. No. A32794, Invitrogen), and goat polyclonal anti-rabbit IgG linked to Alexa Fluor™ 488 (Cat. No. A-11,008, Invitrogen).

### qRT-PCR analysis

Total RNA was isolated using Trizol (Cat. No. 15,596,026, Invitrogen). Reverse transcription into cDNA was carried out using the PrimeScript™ RT Master Mix (Cat. No. RR036A, Takara) and the miRNA primer set according to the manufacturer’s instructions. qRT-PCR was performed on an ABI 7500 Real-Time PCR system (Applied Biosystems, CA, USA). The reverse transcription protocol for miRNA or mRNA cDNA synthesis included incubation at 26 °C for 40 min, 42 °C for 40 min, 85 °C for 10 min, and preservation at 4 °C; followed by 42 °C for 45 min, 85 °C for 5 min, and preservation at 4 °C. GAPDH was used for mRNA expression normalization. Amplification was carried out using iTaq™ Universal SYBR® Green Supermix (Cat. No. 1,725,124, Bio-Rad) with denaturation at 95 °C, followed by 40 cycles of denaturation at 95 °C for 15 s and annealing at 60 °C for 1 min. Relative expression of the target genes was determined using the 2-ΔΔCq method. Specific primer sequences were as follows: GAPDH (forward: GGTTGTCTCCTGCGACTTCA, reverse: TGGTCCAGGGTTTCTTACTCC), acta2(forward: TCGCTGGTGATGATGCT, reverse: TGGTGATGATGCCGTGT), fibronectin1(FN1) (forward: GACCCTTACACGGTTTCCCA, reverse: TGGCACCATTTAGATGAATCGC), collagen1(col1) (forward: CGATGGATTCCCGTTCGAGT, reverse: GAGGCCTCGGTGGACATTAG), lysyloxidase(lox) (forward: GATTGCCACAAGATTTCCA, reverse: TTCCCTTTCCCTTTCCC), miR-27b-3p (forward: AATGGCGTTCACAGTGGCTAAG, reverse: GTGCAGGGTCCGAGGT).

### Western blots

The proteins of FAP were extracted using a lysis mixture composed of protease inhibitor, phosphatase inhibitor (Cat. No. P1010, Beyotime), and RIPA lysis buffer (Cat. No. P0013C, Beyotime) in a ratio of 1:1:25. The cell lysis process was carried out on ice. The protein concentration was quantified using a BCA protein assay kit (Cat. No. P0012S, Beyotime). Subsequently, 30 µg of protein-containing samples were loaded onto a 10% SDS-PAGE gel and transferred to a polyvinylidene difluoride (PVDF) membrane (Cat. No. 1,620,177, Bio-Rad Laboratories). The membrane was blocked with 5% BSA in PBS and then incubated with the primary antibody, which was diluted in 1% BSA, overnight at 4 °C. Following this, the membrane was exposed to an HRP-conjugated secondary antibody, diluted in 1% BSA, for 1 h at room temperature. The signal was visualized using a ChemiDoc Touch Imaging System scanner (Bio-Rad Laboratories, CA, USA). Details of the primary and secondary antibodies utilized: TGF-βR1 primary antibody (Cat. No. AF5374, Affinity), Smad2/3 antibody (Cat. No. AF6367, Affinity), P-smad2/3 antibody (Cat. No. bs-8853R, Bioss), HRP-conjugated Goat Anti-Rabbit IgG (Cat. No. SA00001-2, Proteintech).

### Statistical analysis

All data are presented as mean ± SE. Independent samples t-test was used for the comparison between two groups, and one-way analysis of variance (ANOVA) was used for the comparison among more than two groups, and Tukey post hoc test is used for multiple comparisons between groups. Statistical significance was defined as *p* < .05, *p < .05, **p < .01. Each experiment was repeated at least three times.

## RESULTS

### FAPs exhibit fibrotic phenotype in denervated muscle

**Fig. 1:**
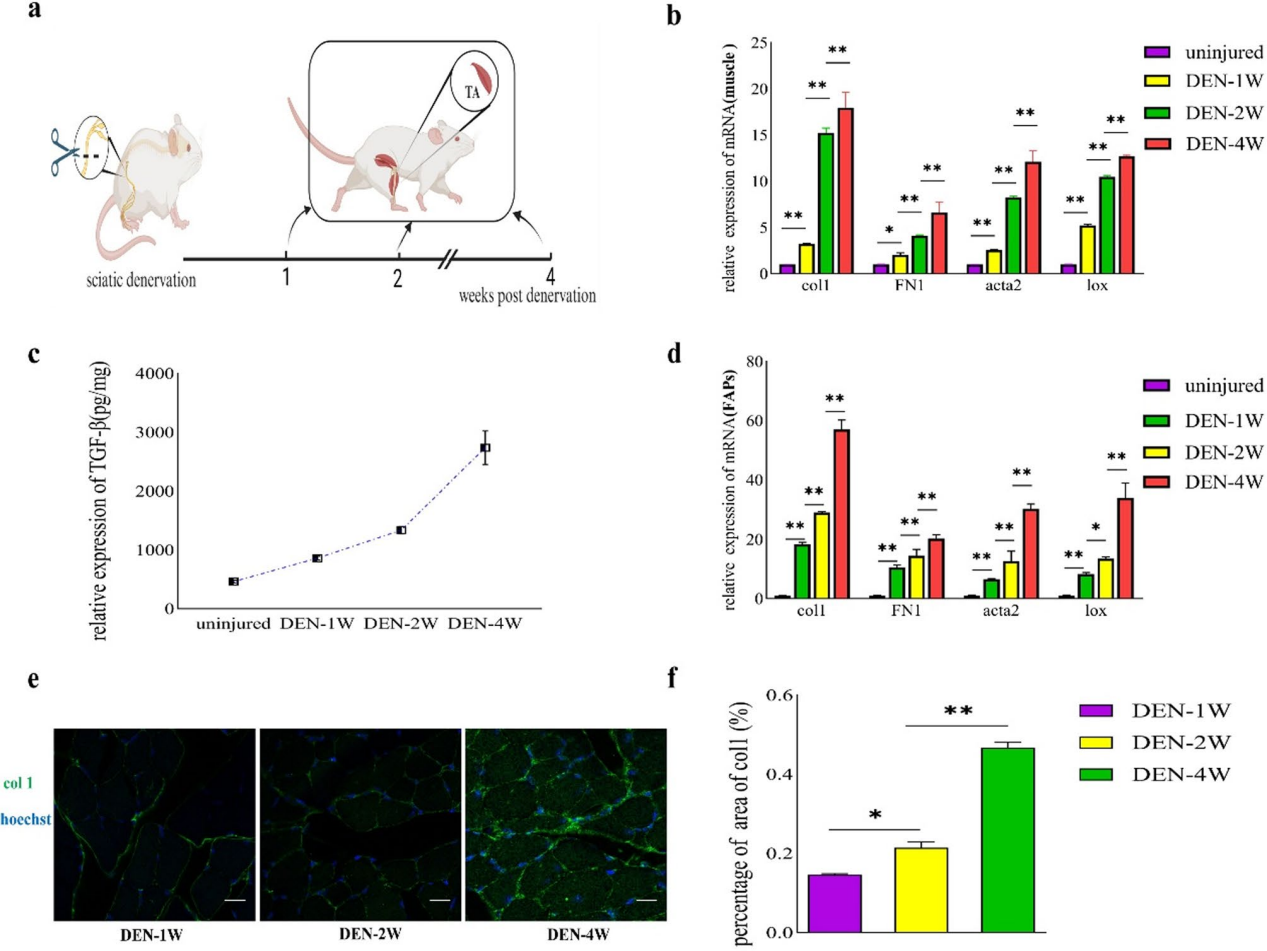
FAPs derived from denervated muscle exhibit fibrotic phenotype. **a**, a schematic showing the experiment: collected TA at DEN-1 W, DEN-2 W, DEN-3 W and DEN-4 W for further analysis. **b**, the mRNA levels of fibrogenic markers in uninjured muscles and denervated muscles were analyzed by qRT-PCR. **c**, concentration of TGF-β in uninjured muscles and denervated muscles were tested by TGF-β ELISA assays. **d**, the mRNA levels of fibrogenic markers of purified FAPs isolated from uninjured muscles and denervated muscles were examined by qRT-PCR. **e, f**, immunofluorescence for col 1 of TA collected from denervated muscles and the percentage of area of col1, scale bar,100 μm. All data are represented as mean ± SEM (*n* = 3). *P < .05, **P < .01

To investigate the mechanism by which FAPs differentiate into myofibroblasts during chronic skeletal muscle injury, we developed a model with sciatic nerve transected in C57BL/6 mice. TA were collected at DEN-1 W, DEN-2 W, and DEN-4 W individually (Fig. 1a). The ECM mainly consists of various collagens, elastin, non-collagen glycoproteins, and lox that catalyzes covalent cross-link formation in collagen and elastin. Myofibroblasts expressing alpha-smooth muscle actin (α-SMA, acta2) are the primary ECM producers [30, 31]. Results from qRT-PCR analysis of TA from mice at DEN-1 W, DEN-2 W, and DEN-4 W showed a progressive increase in mRNA expression of acta2, FN1, lox and col1 compared to uninjured mice. Particularly, col1 mRNA expression at DEN-4 W was significantly elevated, approximately 20-fold higher than in uninjured mice (Fig. 1b). Immunofluorescent staining of tissue sections also demonstrated a corresponding rise in col1 deposition with prolonged denervation (Fig. 1e and f, supplementary Fig.  1d).

TGF-β is a potent pro-fibrotic factor that plays a crucial role in the fibrotic processes of various organs [32–34]. We evaluated the weight of TA and TGF-β levels in uninjured mice and mice at different time points following nerve denervation, revealing a gradual increase in TGF-β concentration in the muscle (Fig. 1c).

To further understand the fibrotic characteristics of muscle fibers post-denervation, FAPs were isolated from the TA at DEN-1 W, DEN-2 W, DEN-4 W and uninjured mice according to the method described. We observed a rise in the proportion of FAPs with the duration of denervation (supplementary Fig.  1b,  1c), consistent with findings by Luca Madaro et al. [35]. qRT-PCR analysis of primary FAPs also demonstrated a progressive increase in mRNA expression of acta2, FN1, lox, and col1, with col1 mRNA expression at DEN-4 W exhibiting the most significant elevation, approximately 60 times higher than in FAPs from uninjured mice (Fig. 1d). These findings collectively suggested the emergence of a fibrotic phenotype in FAPs from mice with nerve transected.

### miR-27b-3p negatively regulates fibrogenic differentiation of FAPs in vitro

**Fig. 2:**
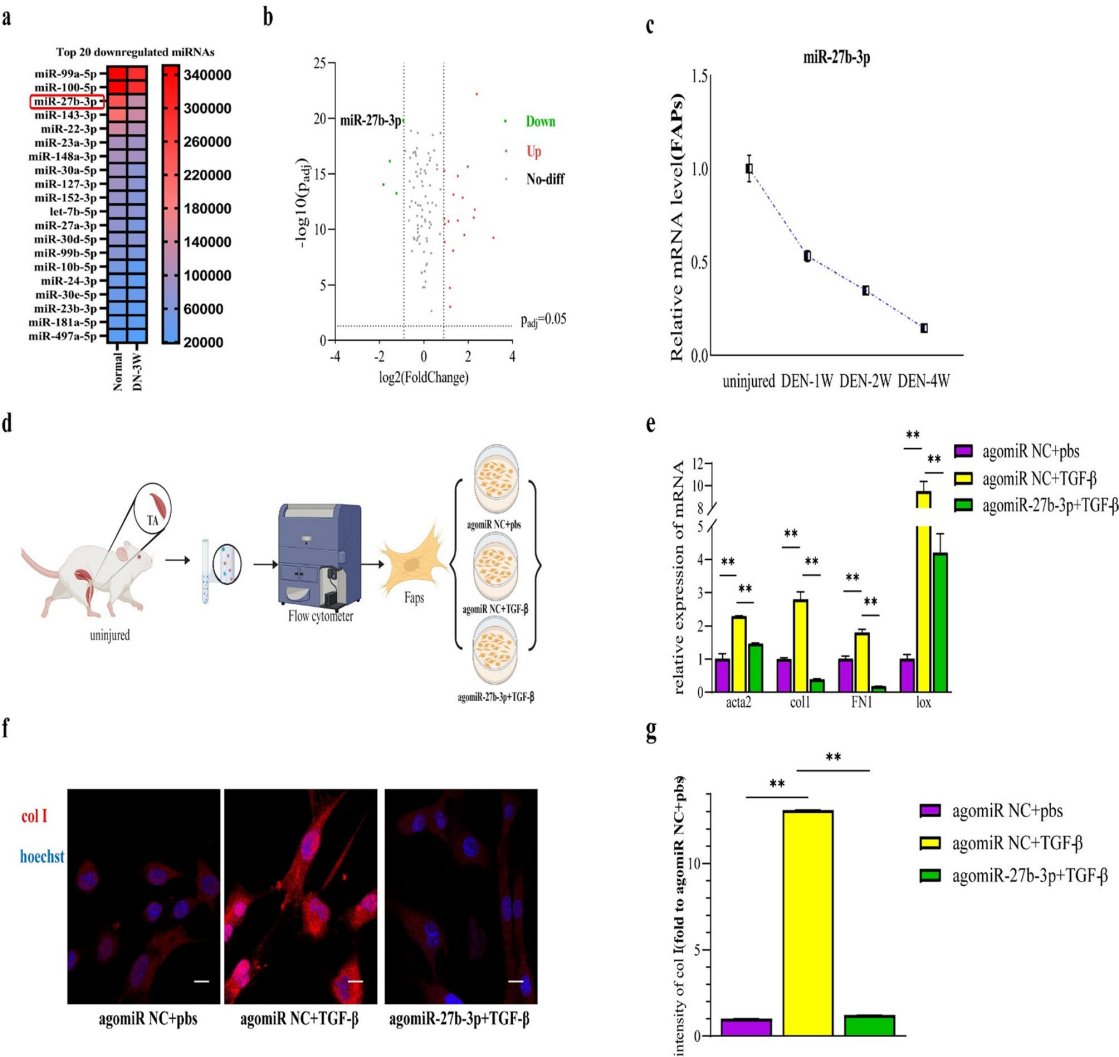
miR-27b-3p efficiently decreases fibrogenic differentiation of FAPs in vitro. **a, b**, heat map and volcano plots showed the miRNAs with different levels between FAPs isolated from DEN-3 W and uninjured mice. **c**, relative expression of miR-27b-3p in purified FAPs isolated from uninjured muscles and denervated muscles. **d**, a schematic showing the experiment in vitro. **e**, the level of acta2, FN1, lox and col1 in FAPs induced by TGF-β and combined transfection with agomiR-27b-3p or agomiR NC were analyzed by qRT-PCR. **f, g**, Immunofluorescence of col1 and intensity of col 1. scale bar, 100 μm. All data are represented as mean ± SEM (*n* = 3). *P < .05, **P < .01

MiRNAs play a pivotal role in modulating gene expression, cell proliferation, and differentiation. In the realm of orthopedics and sports medicine, miRNAs also exert influence on conditions such as osteoporosis, rheumatoid arthritis, and the diagnosis and treatment of tendon injuries, among others [36–40]. Recent studies have highlighted the impact of miRNAs on differentiation of FAPs. To investigate the significant influence of miRNAs on the fibrotic differentiation of FAPs during chronic skeletal muscle injury, we compared the miRNA expression profiles of primary FAPs from uninjured mice and those from DEN-3 W. Analysis revealed a marked reduction in the abundance of miR-27b-3p in FAPs from DEN-3 W compared to those from uninjured mice, as depicted in the cluster heatmap and volcano plot (Fig. 2a and b). Promisingly, miR-27b-3p plays a critical role in the pathogenesis of fibrosis in the lungs, kidneys, and heart. Elevating its expression levels can significantly attenuate fibrotic processes in these organs.

Furthermore, qRT-PCR analysis at different time points also confirmed a significant decrease in miR-27b-3p levels (Fig. 2c). Additionally, Nicoletta et al. utilized Nitric Oxide (NO) to mitigate the disease progression in mdx-mice, an animal model of Duchenne muscular dystrophy (DMD), by increasing the abundance of miR-27b [41]. This suggests that miR-27b-3p may have a role in inhibiting fibrosis of muscle.

To test this hypothesis, we isolated FAPs from uninjured muscles and induced fibrosis by administering recombinant TGF-β protein, a cytokine known to induce fibrosis in various experiments. Subsequently, we treated the cells with agomiR-27b-3p or agomiR NC (Fig. 2d). Prior to treatment, we ensured the safety of agomiR-27b-3p and agomiR NC. CCK-8 assay revealed that the concentrations of these agents used in our study were safe within the 72-hour timeframe required for the experiment (supplementary Fig.  2a). And qRT-PCR results demonstrated that TGF-β upregulated the mRNA levels of acta2, FN1, lox, and col1. However, the addition of agomiR-27b-3p effectively reversed fibrosis, leading to a significant reduction in their expression levels (Fig. 2e).

Furthermore, cell immunofluorescence indicated that the incremental expression of col1 induced by TGF-β was significantly reduced by agomiR-27b-3p (Fig. 2f and g). It is important to note that due to the low level of miR-27b-3p in uninjured mice, the use of inhibitor of miR-27b-3p would not significantly affect fibrosis. In a word, miR-27b-3p can reduce fibrogenic differentiation of FAPs in vitro.

### Treatment of agomiR-27b-3p inhibits muscle fibrosis in denervated mice

**Fig. 3:**
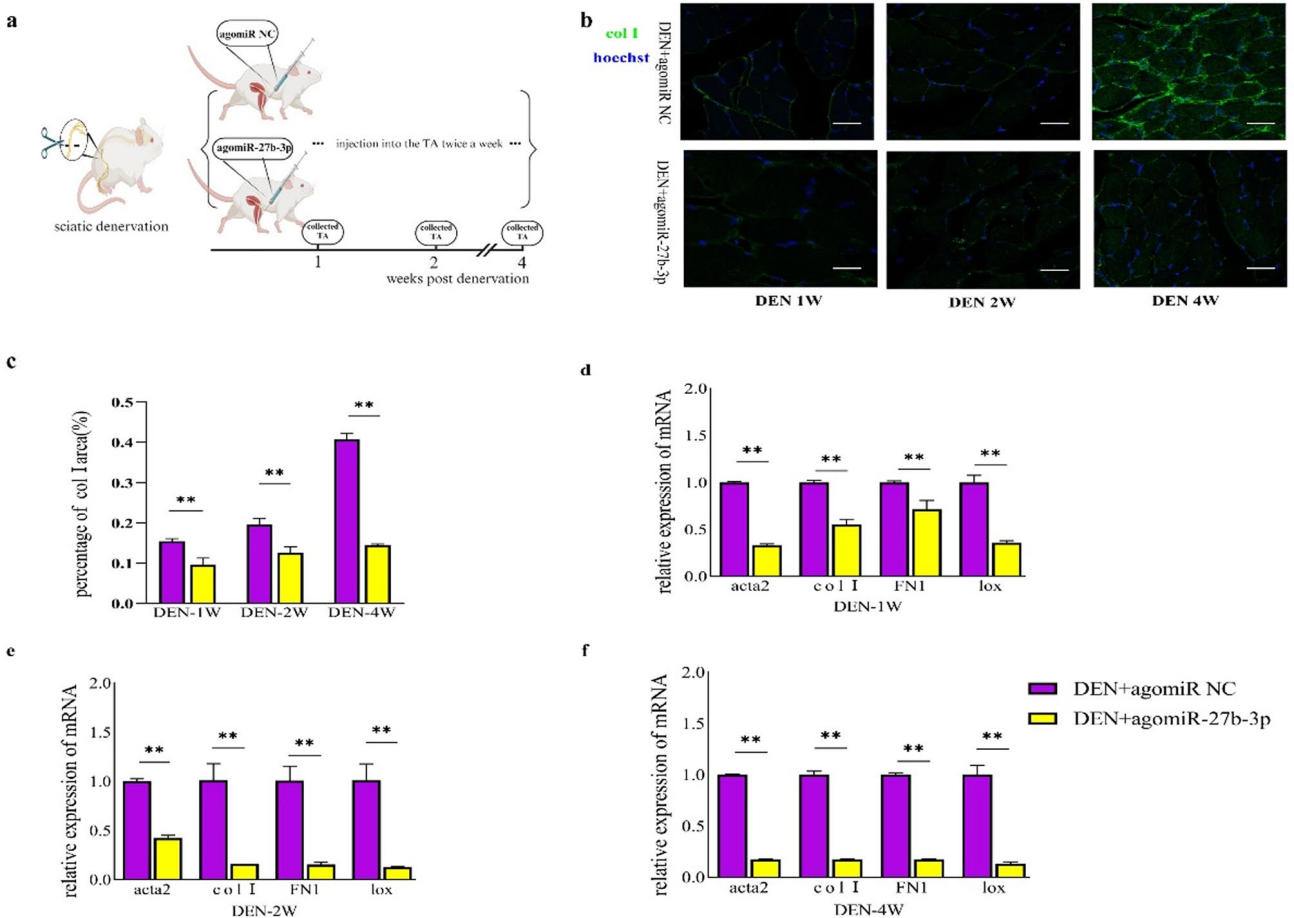
agomiR-27b-3p suppresses muscle fibrosis in denervated mice. **a.** a schematic showing the experiment: injected agomiR NC or agomiR-27b-3p in denervated TA twice a week. **b, c**, Immunofluorescence for col1 of TA collected from denervated mice treatmented with agomiR NC or agomiR-27b-3p, and the percentage of area of col1, scale bar, 100 μm. **d, e, f**, relative expression of fibrogenic markers, acta2, FN1, lox and col1 in purified FAPs isolated from TA treatmented with agomiR NC or agomiR-27b-3p at different time points were examined by qRT-PCR. The legends of the three figures are consistent and are shown in the figure f. All data are represented as mean ± SEM (*n* = 3). *P < .05, **P < .01

To further confirm the inhibitory role of miR-27b-3p in fibrosis, we administered local injections of agomiR-27b-3p and agomiR NC into the TA twice a week following denervation (Fig. 3a). Immunofluorescence analysis of the tissues revealed that the area percentage of col1 in mice receiving agomiR NC steadily increased over time, whereas mice injected with agomiR-27b-3p exhibited a reduction in the col1 area percentage, thereby delaying the fibrotic process (Fig. 3b and c, and supplementary Fig.  2c). Furthermore, qRT-PCR results demonstrated that agomiR-27b-3p notably decreased the levels of acta2, FN1, lox, and col1 compared to mice injected with agomiR NC (Fig. 3d, e and f). Hence, we could conclude that miR-27b-3p plays an anti-fibrotic role in vivo as well.

### TGF-βR1 is the target gene of miR-27b-3p and regulates the fibrogenic capacity of FAPs

**Fig. 4:**
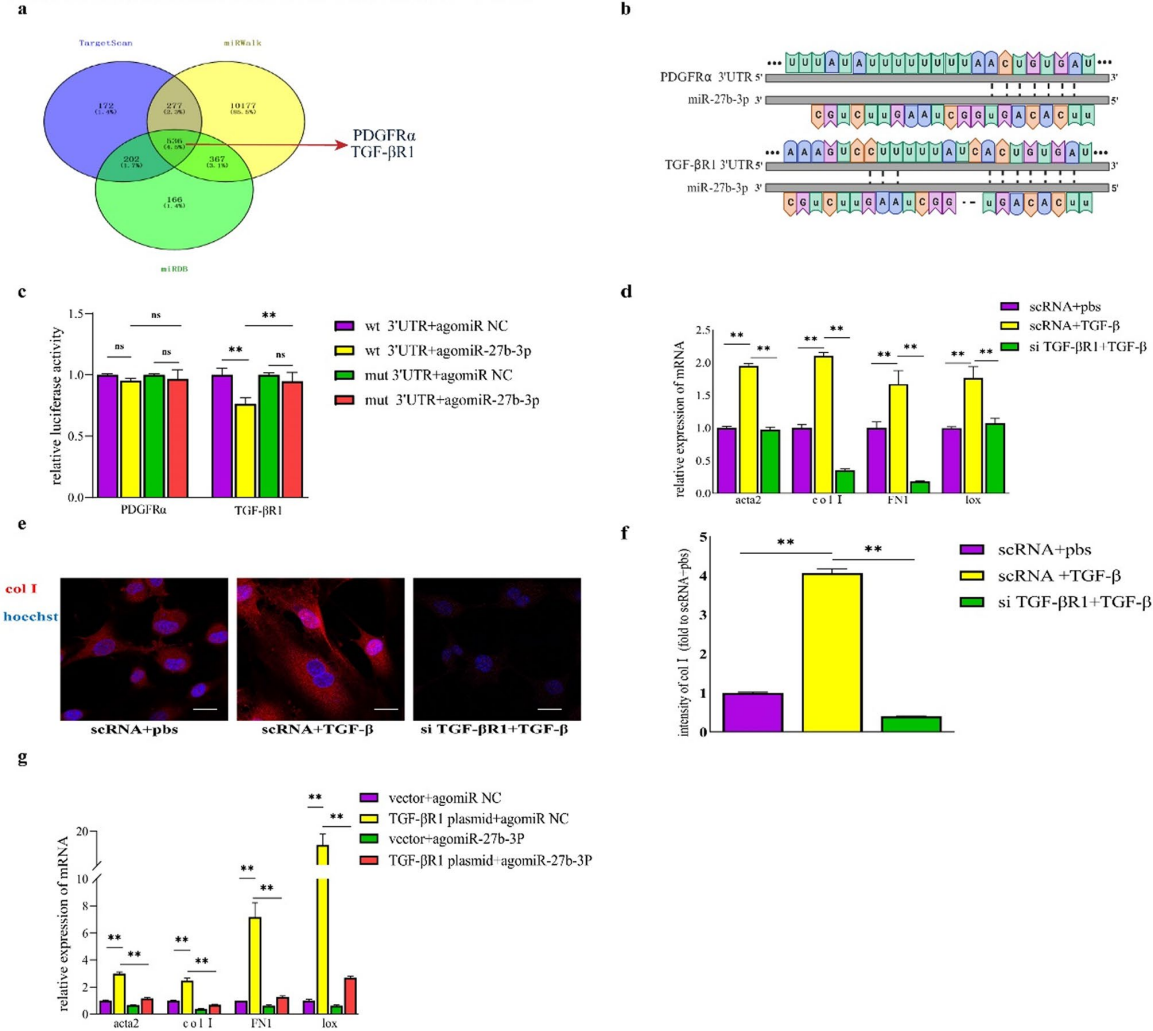
TGF-βR1 is the target gene of miR-27b-3p and regulates the fibrogenic capacity of FAPs. **a**, Bioinformatics analysis was used to predict the potential target genes of miR-27b-3p. **b**, A schematic showing predicted binding site of miR-27b-3p and the 3’UTR of candidate target genes’ mRNAs. **c.** The luciferase activity in 293T cells was tested using the Dual-Luciferase Reporter System. **d**, the expressions of fibrogenic genes in TGF-β-induced FAPs transfected with scRNA or si TGF-βR1. **e, f**, Immunofluorescence for col1 and average intensity of col1 in TGF-β-induced FAPs transfection with scRNA or si TGF-βR1, scale bar,100 μm. **g**, the mRNA expressions of acta2, FN1, lox and col1 in freshly purified Faps combined treatment with or without si TGF-βR1 plus plasmid with high expression of TGF-βR1 were examined by qRT-PCR. All data are represented as mean ± SEM (*n* = 3). *P < .05, **P < .01

To further elucidate the mechanisms through which miR-27b-3p exerts its anti-fibrotic effects, we employed bioinformatics tools such as TargetScan, miRDB and miRWalk to predict potential target genes of miR-27b-3p. The computational analysis suggested that platelet-derived growth factor receptor-alpha (PDGFRα) and TGF-βR1 could be targets of miR-27b-3p, along with their respective binding sites (Fig. 4a and b). Then, we conducted dual-luciferase reporter assays to validate these predictions. Interestingly, when the binding site of PDGFRα was mutated, there was no significant difference observed between the transfection of agomiR-27b-3p and agomiR NC. In contrast, when the binding site of TGF-βR1 was mutated, the significant difference between transfection with agomiR-27b-3p and transfection with agomiR NC in WT mice disappeared (Fig. 4c). This observation confirmed the specific binding of miR-27b-3p to TGF-βR1.

Subsequently, we conducted separate transfections with si TGF-βR1 and plasmids overexpressing TGF-βR1 to investigate the influence of TGF-βR1 expression on the fibrogenic potential of FAPs. The qRT-PCR analysis revealed that the upregulation of acta2, FN1, lox and col1 induced by TGF-β was significantly attenuated by efficient transfection of si TGF-βR1 (Fig. 4d, supplementary Fig.  2d). The induction of TGF-β resulted in a nearly 4-fold increase in the intensity of col1 compared to group transfected with sc RNA and added with PBS, while successful transfection of si TGF-βR1 reduced the intensity of col1 to approximately 40.0% of the control group (Fig. 4e and f). Furthermore, transfection with a plasmid overexpressing TGF-βR1 demonstrated that increased TGF-βR1 levels resulted in elevated mRNA levels of acta2, FN1, lox and col1. Nevertheless, the application of agomiR-27b-3p reduced the expression of acta2, FN1, lox and col1 to levels ranging from 14.1 to 39.0% (Fig. 4g).

In conclusion, the aforementioned findings suggested a specific interaction between TGF-βR1 and miR-27b-3p, and the expression levels of TGF-βR1 align with the fibrogenic potential of FAPs.

### miR-27b-3p regulates TGF-βR1/Smad pathway

TGF-β is a multifunctional cytokine comprising isoforms TGF-β1–3, which has earned the title of “primary regulator of fibrosis”. The TGF-β signaling pathway plays a substantial role in pathogenesis of fibrosis of various organs [33]. Binding of the TGF-β ligand to the Transforming Growth Factor Beta Receptor 2 (TGF-βR2) induces phosphorylation of TGF-βR2, then phosphorylated TGF-βR2 recruits and induces phosphorylation of TGF-βR1 to form a complex. The receptor complex activates downstream Smad proteins, with Smad3 being the most crucial protein in this signal pathway. The level of Smad3 phosphorylation reflects the activation of the TGF-β signaling pathway [34].

**Fig. 5:**
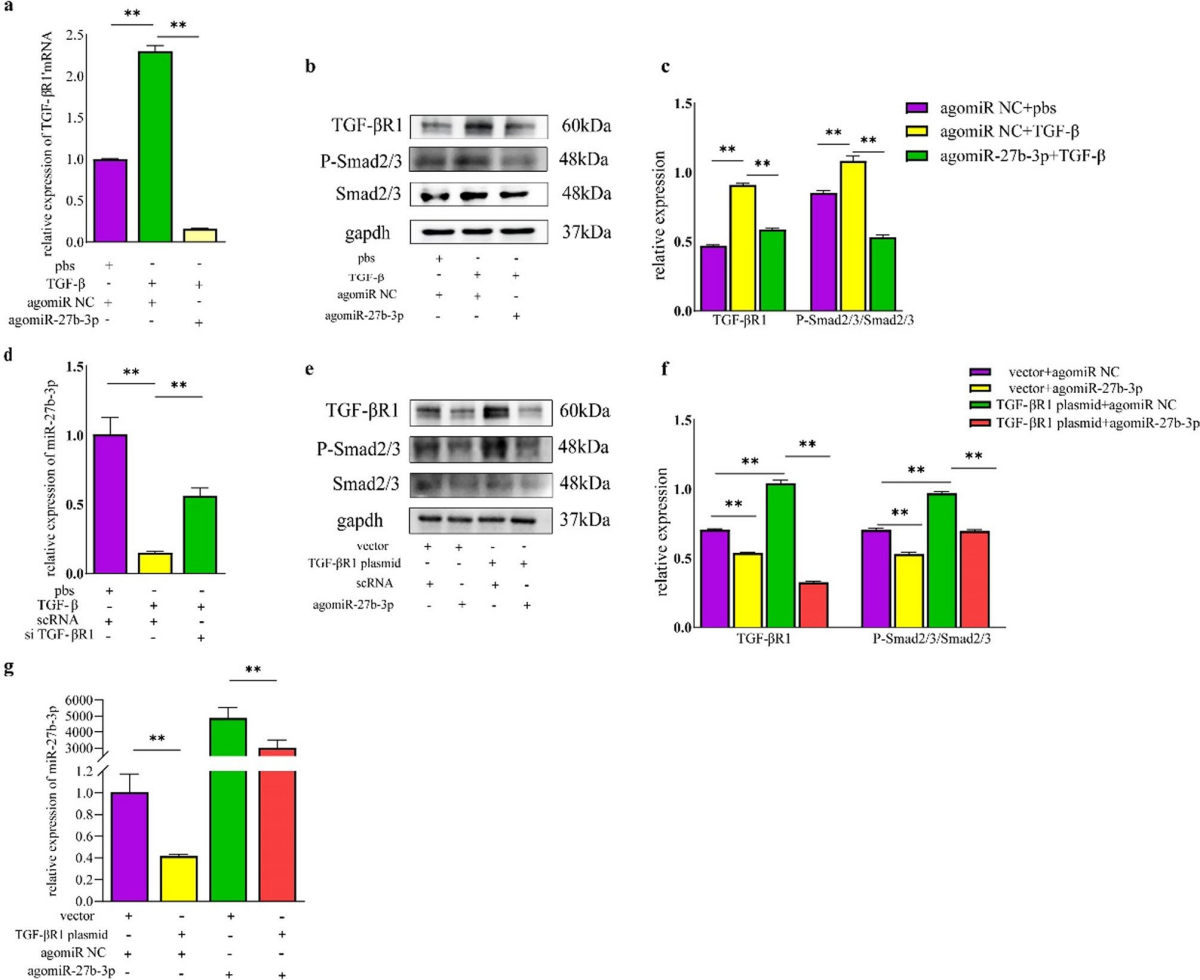
miR-27b-3p regulates fibrogenic capacity of FAPs through TGF-βR1/Smad pathway. **a**, mRNA expressions of TGF-βR1 in FAPs induced by TGF-β and followed by transfection with agomiR-27b-3p or agomiR NC. **b, c**, the protein expression of TGF-βR1, P-Smad2/3 and Smad2/3 in FAPs induced by TGF-β and transfected with agomir-27b-3p or agomiR NC were assayed by WB, and the relative intensity of ratio of TGF-βR1 and P-smad2/3/Smad2/3 were quantified. **d**, relative expression of miR-27b-3p in FAPs transfected with sc RNA or si TGF-βR1. **e, f**, the protein expression of TGF-βR1, P-Smad2/3 and smad2/3 in FAPs combined treatment with agomiR NC or agomiR-27b-3p plus plasmid with overexpression of TGF-βR1 were tested by WB, and the relative intensity of TGF-βR1 and the ratio of P-smad2/3/Smad2/3 were quantified. **g**, the abudance of miR-27b-3p in FAPs combined treatment with agomiR NC or agomiR-27b-3p plus plasmid with overexpression of TGF-βR1. All data are represented as mean ± SEM (*n* = 3). *P < .05, **P < .01

The results we have already described have demonstrated that miR-27b-3p targeted TGF-βR1 and regulated differentiation of FAPs into myofibroblasts. Based on this, we have reason to speculate that miR-27b-3p may regulate fibrosis through the TGF-βR1/Smad pathway. To investigate this further and validate our hypothesis, FAPs from uninjured muscle were isolated and induced fibrosis using recombinant TGF-β protein, followed by efficient transfection with agomiR-27b-3p or agomiR NC (supplementary Fig.  2d). Result of qRT-PCR showed that transfection with agomiR-27b-3p significantly reduced mRNA expression of TGF-βR1 (Fig. 5a). And result of WB also demonstrated that induction of TGF-β increased the expression of TGF-βR1 and the level of phosphorylation of Smad2/3, however, transfecting with agomiR-27b-3p successfully decreased the above elevated trend to 64.5% and 50.0% of the levels induced by TGF-β respectively (Fig. 5b and c).

Moreover, we observed a negative correlation between expression of TGF-βR1 and the abundance of miR-27b-3p. Specifically, transfection with si TGF-βR1 led to an increase in the abundance of miR-27b-3p, while transfection with the plasmid with overexpression of TGF-βR1 decreased level of miR-27b-3p (Fig. 5d and g). Importantly, WB demonstrated that transfection with plasmid with overexpression of TGF-βR1 increased the expression of TGF-βR1 and level of phosphorylation of Smad2/3, However, followed by transfection with agomiR-27b-3p could effectively reduce the above change (Fig. 5e and f). Taken together, miR-27b-3p regulates differentiation of FAPs to myofibroblasts through the TGF-βR1/Smad signaling pathway.

### 6TGF-βR1/Smad negatively regulates the expression of miR-27b-3p

Our previously mentioned findings have demonstrated that miR-27b-3p regulates fibrogenic differentiation of FAPs by targeting the TGF-βR1/Smad signaling pathway, and there is an association between the expression level of TGF-βR1 and abundance of miR-27b-3p. To further validate this signaling pathway and investigate the relationship between expression of downstream molecule and abundance of miR-27b-3p, SIS3, a Smad3 phosphorylation inhibitor was utilized.

**Fig. 6:**
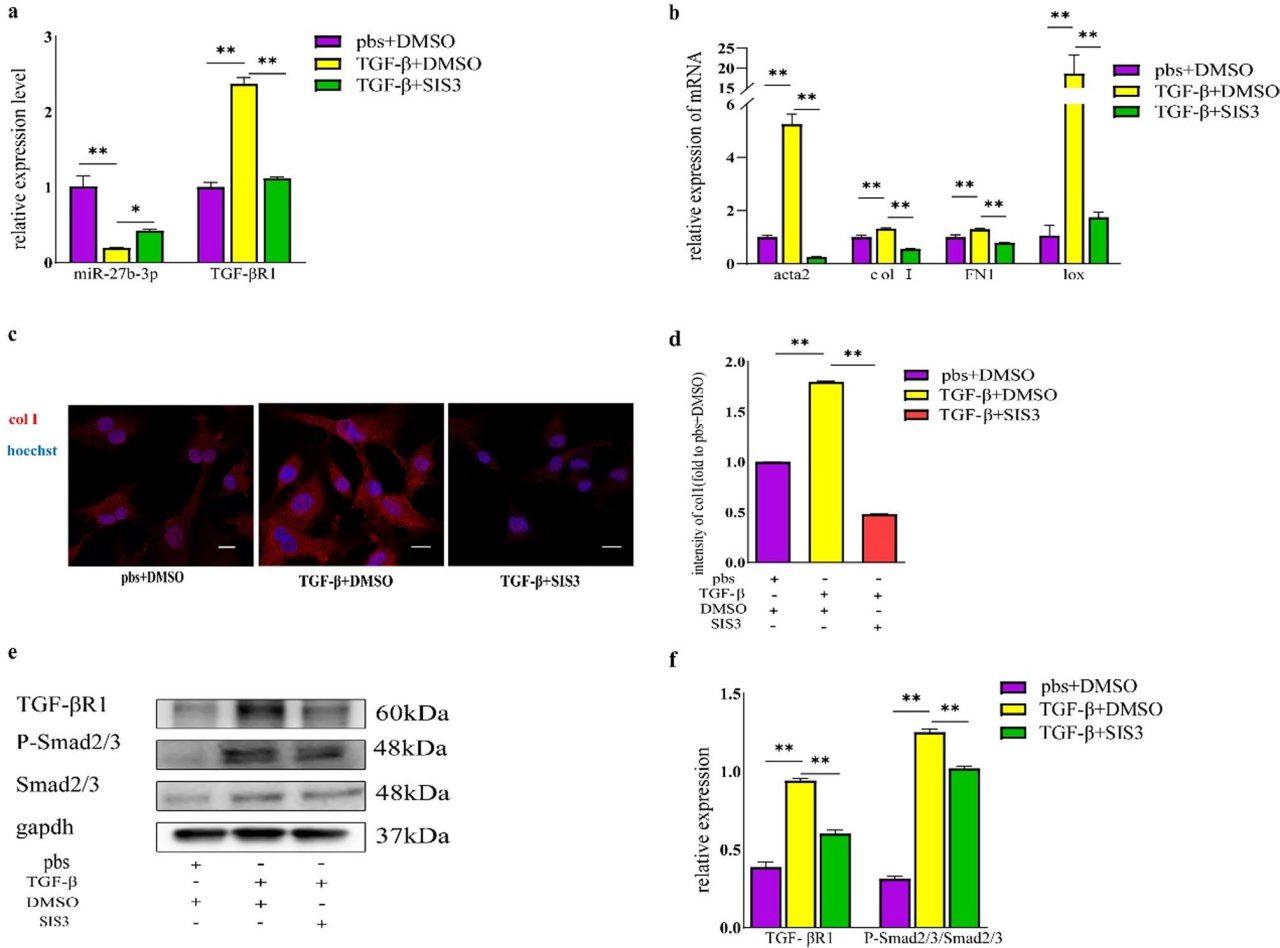
TGF-βR1/Smad negatively regulates the abundance of miR-27b-3p. **a, b**, the mRNA expressions of miR-27b-3p, TGF-βR1, acta2, FN1, lox and col1 in FAPs induced by TGF-β and followed by treatment with or without SIS3 in vitro were examined by qRT-PCR. **c, d**, Immunofluorescence for col1 and average intensity of col1 showed the fibrosis level in FAPs induced by TGF-β and followed by treatment with or without SIS3, scale bar, 100 μm. **e, f**, the protein expression of TGF-βR1, P-Smad2/3 and Smad2/3 in FAPs induced by TGF-β and followed by treatment with or without SIS3, and the relative intensity of TGF-βR1 and the ratio of P-Smad2/3/Smad2/3 were quantified. All data are represented as mean ± SEM (*n* = 3). *P < .05, **P < .01

Result of qRT-PCR showed a significant increase in abundance of miR-27b-3p and decrease in mRNA expression of TGF-βR1 in FAPs added with SIS3 compared to that induced by TGF-β (Fig. 6a). Furthermore, there was an effective reduction in mRNA expression of acta2, FN1, lox, and col1, and mRNA expression of acta2 showed the most significant decrease to only 5.0% of the FAPs induced by TGF-β (Fig. 6b). Consistent with results of qRT-PCR, cell immunofluorescence also demonstrated that SIS3 effectively reduced the intensity of col1 to only 26.8% of the FAPs induced by TGF-β (Fig. 6c and d). Result of WB further showed that SIS3 significantly reduced levels of phosphorylation of smad2/3 and protein expression of TGF-βR1(Fig. 6e and f).

**Fig. 7:**
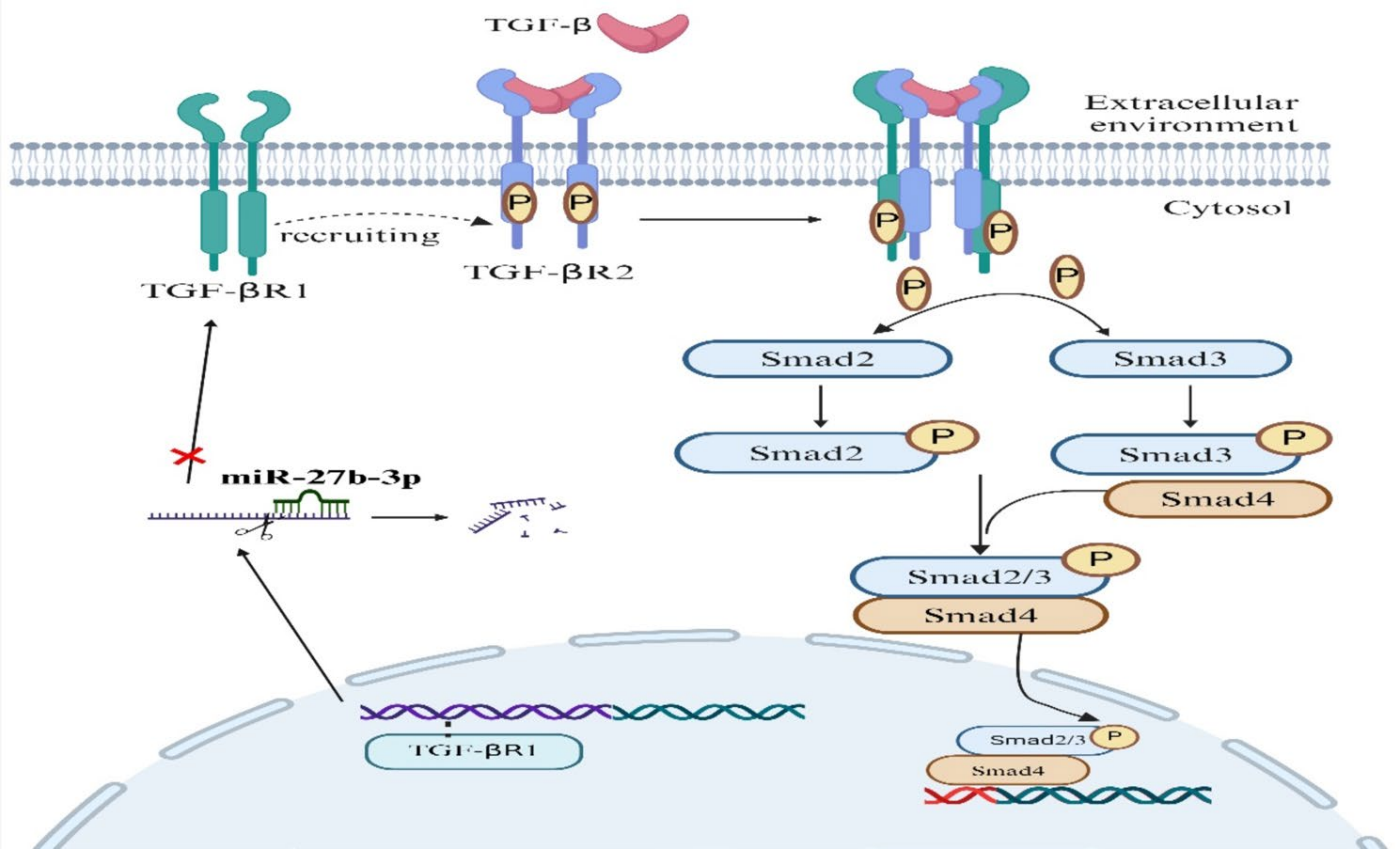
Schematic diagram of miR-27b-3p/TGF -βR1/Smad axis in fibrogenic differentiation of FAPs

In conclusion, level of phosphorylation of Smad2/3 and expression of TGF-βR1 can negatively regulate abundance of miR-27b-3p, and miR-27b-3p regulates fibrogenic differentiation of FAPs by targeting TGF-βR1/Smad signaling pathway (Fig. 7).

## Discussion

Skeletal muscle injuries account for up to 50% of all sports-related injuries. These injuries can result from direct mechanical trauma or indirect causes such as vascular and neurological dysfunction [42]. Following a muscle injury, satellite cells within the muscle are activated, initiating processes like proliferation, differentiation, and fusion to form regenerated muscle fibers, showcasing the muscle’s robust regenerative capacity [43, 44]. However, effective muscle regeneration necessitates intricate interactions among satellite cells, endothelial cells, immune cells, FAPs, and various cytokines secreted by non-muscular tissue-derived cells [45].

Previous research has highlighted that FAPs secrete a range of cytokines including insulin-like growth factor 1 (IGF1), interleukin-6 (IL-6), interleukin-15 (IL-15), interleukin-10 (IL-10), and inhibin, among others, which play direct or indirect roles in promoting satellite cell proliferation [3, 32, 46, 47]. Studies have also indicated that either AMPKα1 overexpression or exercise can enhance FAPs activity, thereby improving muscle regeneration [48, 49]. FAPs possess the ability to differentiate into myofibroblasts and serve as a significant source of ECM, crucial for providing structural support during muscle repair. Consequently, FAPs are recognized as pivotal regulatory elements influencing satellite cell function and skeletal muscle regeneration [50].

In cases of chronic skeletal muscle injury, FAPs persist and continue to differentiate, resulting in excessive deposition of ECM. This excessive ECM deposition significantly hinders the space available for muscle fiber regeneration and heightens the risk of further injury [7, 45]. Consistent with the observations made by Luca Madaro and colleagues, our study noted a progressive increase in the proportion of FAPs in mice with sciatic nerve denervated, accompanied by advancing muscle fibrosis characterized by elevated mRNA expression of col1, FN1, lox, and acta2-markers indicative of myofibroblasts [8]. Furthermore, there was a gradual rise in the deposition of col1, a prominent collagen type in the ECM. Prior research has established that TGF-β plays a pivotal role in driving fibrosis, with TGF-β levels correlating with the progression of fibrotic conditions in various organs such as the liver, lung, kidney, skin and heart [51–53]. In our investigation, we observed a consistent increase in concentration of TGF-β in denervated mice, aligning with the augmented ECM deposition and displaying a time-dependent escalation.

MiRNAs represent a class of highly conserved small non-coding RNAs that exert critical regulatory functions in various pathophysiological processes by either inhibiting translation or facilitating the degradation of target mRNAs through base pairing interactions [54]. This study identified notable distinctions in the expression patterns of miRNAs between uninjured mice and those subjected to denervation. Among these variances, miR-27b-3p emerged as one of the most prominently altered miRNAs. Remarkably, the levels of miR-27b-3p exhibited a progressive decline in denervated mice, underscoring its potential involvement in the fibrotic processes observed in this context.

Interestingly, despite variations in the specific molecular signaling pathways involved, a consistent trend is observed wherein elevating the levels of miR-27b-3p leads to a reduction in fibrosis [22–24]. This effect has been demonstrated across different contexts. For example, genetic elimination of miR-27b has been shown to mitigate pathological cardiac remodeling triggered by transverse aortic constriction, encompassing cardiac hypertrophy, myocardial fibrosis, and inflammation [55]. In liver fibrosis induced by CCL4, miR-27b-3p targets the Yes-associated protein (YAP)/Lysyloxidase-like2 (LOXL2) pathway, effectively slowing down the progression of fibrosis [56]. Moreover, miR-27b-3p has been found to degrade the myostatin gene, leading to enhanced proliferation and inhibited differentiation of primary skeletal muscle cells [57]. Furthermore, miR-27b-3p plays a crucial role in muscle atrophy and the differentiation of skeletal muscle myoblasts (C2C12) [58].

In line with these findings, transfection with agomiR-27b-3p in vitro effectively reduced fibrosis induced by TGF-β in our study. Moreover, local injection of agomiR-27b-3p in denervated tibialis anterior muscles resulted in a significant decrease in the mRNA expression of col1, FN1, acta2, and lox, along with reduced col1 deposition at various time points following denervation. In summary, agomiR-27b-3p has the potential to inhibit the differentiation of FAPs into myofibroblasts.

It’s known that TGF-β signaling pathway plays a substantial role in pathogenesis of fibrosis, and the Smad complex binds to DNA and regulates the transcription of target genes [34]. Indeed, researchers have demonstrated that the usage of antibodies against latent TGF-β-binding protein 4 (LTBP4) can decrease LTBP4 proteolytic cleavage, thereby reducing release of TGF-β and alleviating muscle fibrosis in patients with DMD [59]. In addition, tyrosine kinase inhibitors such as Nilotinib and FDA-approved drugs like sunitinib and losartan inhibited differentiation of FAPs to myofibroblasts and alleviated fibrosis by blocking TGF-β signaling pathway [60, 61]. Encouragingly, our study confirmed that miR-27b-3p could specifically target and bind to TGF-βR1. The expression of TGF-βR1 aligned with the deposition of col1 and exhibited a similar trend with the relative mRNA expression of col1, FN1, lox and acta2. However, the transfection with agomiR-27b-3p could diminish the elevated fibrotic capacity of FAPs caused by high level of expression of TGF-βR1. Further investigation revealed that agomiR-27b-3p and si TGF-β reduced the protein and relative mRNA expression of TGF-βR1 and effectively lowered the level of phosphorylation of Smad2/3. Moreover, expression of TGF-βR1 is negatively correlated with the abundance of mir-27b-3p, which corroborated to some extent that miR-27b-3p acted on TGF-βR1.

In our study, we employed a Smad3 phosphorylation inhibitor to investigate the relationship between downstream transcription factors and the levels of miR-27b-3p. By reducing the phosphorylation of Smad2/3, we observed an increase in the abundance of miR-27b-3p and a decrease in the expression of TGF-βR1, thereby reducing fibrosis induced by TGF-β. This reduction in fibrosis was evidenced by decreased mRNA expression of col1, FN1, lox, and acta2, as well as a reduction in col1 deposition. Consequently, we can infer that miR-27b-3p mitigates the fibrogenic differentiation of FAPs by targeting the TGF-βR1/Smad signaling pathway. Furthermore, our findings suggested that TGF-βR1/Smad negatively influenced the abundance of miR-27b-3p.

However, the emergence of single-cell sequencing has shed light on the phenotypic and functional diversity within FAPs subpopulations [62, 63]. Giuliani and colleagues noted that FAPs expressing high levels of sca-1 exhibited a stronger inclination towards adipogenic differentiation in vitro. These cells also displayed increased sensitivity to fibrotic stimuli, resulting in elevated expression of col1 and tissue inhibitor of metalloproteinase1 (TIMP1) [64]. Malecova et al. identified two distinct FAPs subpopulations characterized by the expression of TEK receptor tyrosine kinase (Tie2) and vascular cell adhesion molecule 1 (Vcam1). The Vcam1 + subpopulation was identified as the primary responder to acute injury and displayed pro-fibrotic gene expression traits [65]. Furthermore, Quentin et al. demonstrated that basal cells from subcutaneous adipose tissue contribute to the pool of FAPs [66]. Therefore, investigating the role of miR-27b-3p in specific FAPs subpopulations is crucial for developing more targeted anti-fibrotic strategies. Previous studies have indicated that extracellular matrix (ECM) deposition within 2–4 days post-denervation is correlated with increased levels of connective tissue growth factor (CTGF) [67], while upregulation of the TGF-β signaling pathway occurs one week after denervation. Exploring the interplay between acute and chronic muscle injury processes to analyze signaling pathway crosstalk may unveil novel avenues for effective anti-fibrotic interventions. Additionally, understanding the susceptibility of miRNAs warrants exploration of targeted and degradation-resistant biomaterials.

In summary, despite the diversity among FAPs and potential variations in fibrotic mechanisms during different stages of chronic muscle injury, our research offers new insights into the regulation of muscle fibrosis. MiR-27b-3p acts on the TGF-βR1/Smad signaling pathway to inhibit the differentiation of FAPs into myofibroblasts both in vivo and in vitro, thereby demonstrating an anti-fibrotic effect. Our findings establish a foundational framework for considering miR-27b-3p as a potential therapeutic target to mitigate fibrosis in chronic muscle injury, with implications for clinical application through biotechnological approaches.

### Electronic supplementary material

Below is the link to the electronic supplementary material.

Supplementary Material 1

## Data Availability

No datasets were generated or analysed during the current study.
