# Supplementary material for: miR-27b-3p reduces muscle fibrosis during chronic skeletal muscle injury by targeting TGF-βR1/Smad pathway
Source: J Orthop Surg Res. 2024 Jun 2;19:329. doi: 10.1186/s13018-024-04733-9 (PMC11145862; doi:10.1186/s13018-024-04733-9)
Supplement: Supplementary file 1 — Supplementary Material 1 [file 13018_2024_4733_MOESM1_ESM.docx]

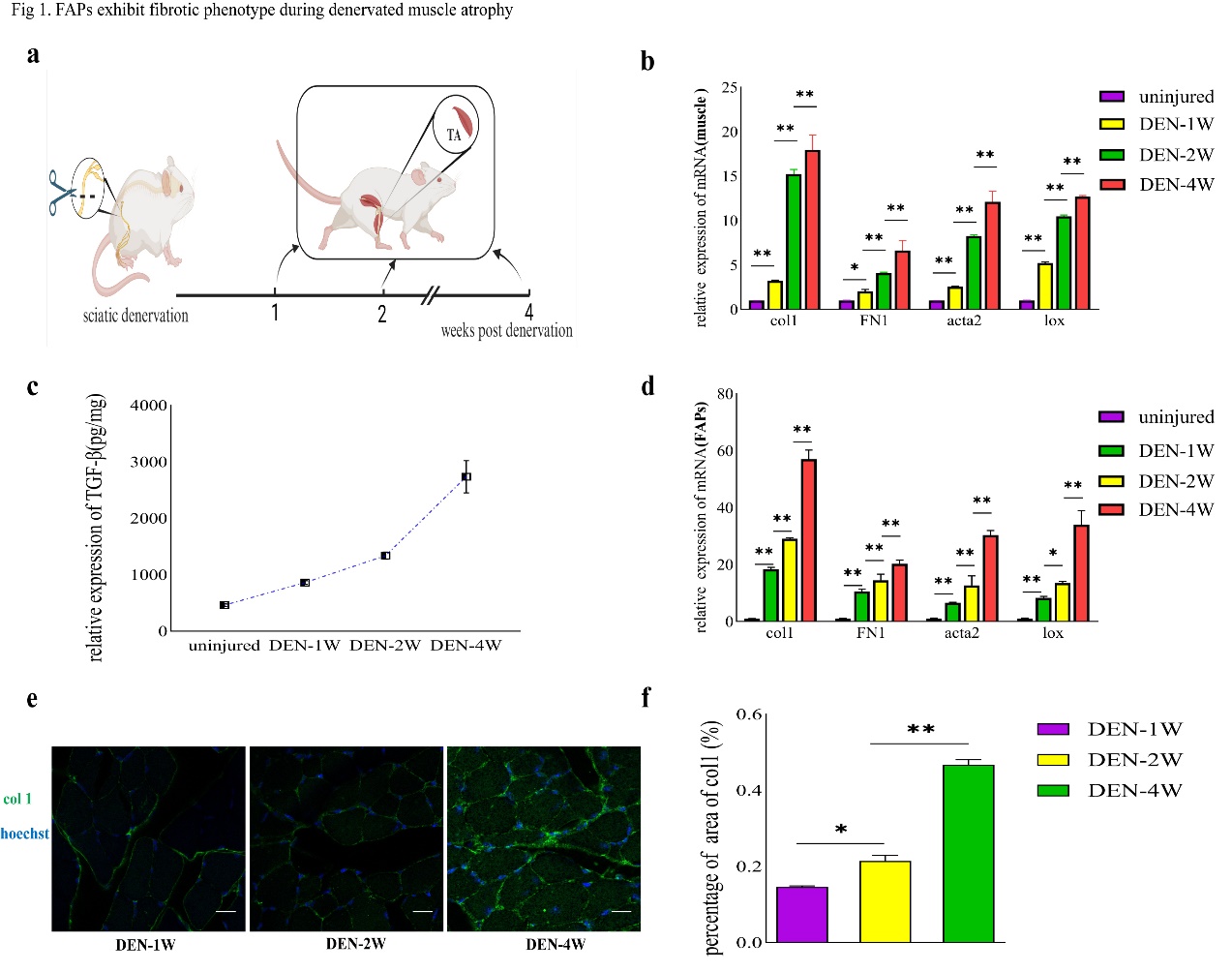


**FIGURE1** FAPs derived from denervated muscle exhibit fibrotic phenotype. **a,** a schematic showing the experiment: collected TA at DEN-1W, DEN-2W, DEN-3W and DEN-4W for further analysis. **b,** the mRNA levels of fibrogenic markers in uninjured muscles and denervated muscles were analyzed by qRT-PCR. **c,** concentration of TGF-β in uninjured muscles and denervated muscles were tested by TGF-β ELISA assays. **d,** the mRNA levels of fibrogenic markers of purified FAPs isolated from uninjured muscles and denervated muscles were examined by qRT-PCR. **e, f,** immunofluorescence for col 1 of TA collected from denervated muscles and the percentage of area of col1, scale bar,100um. All data are represented as mean ± SEM (n = 3). *P < .05, **P < .01.


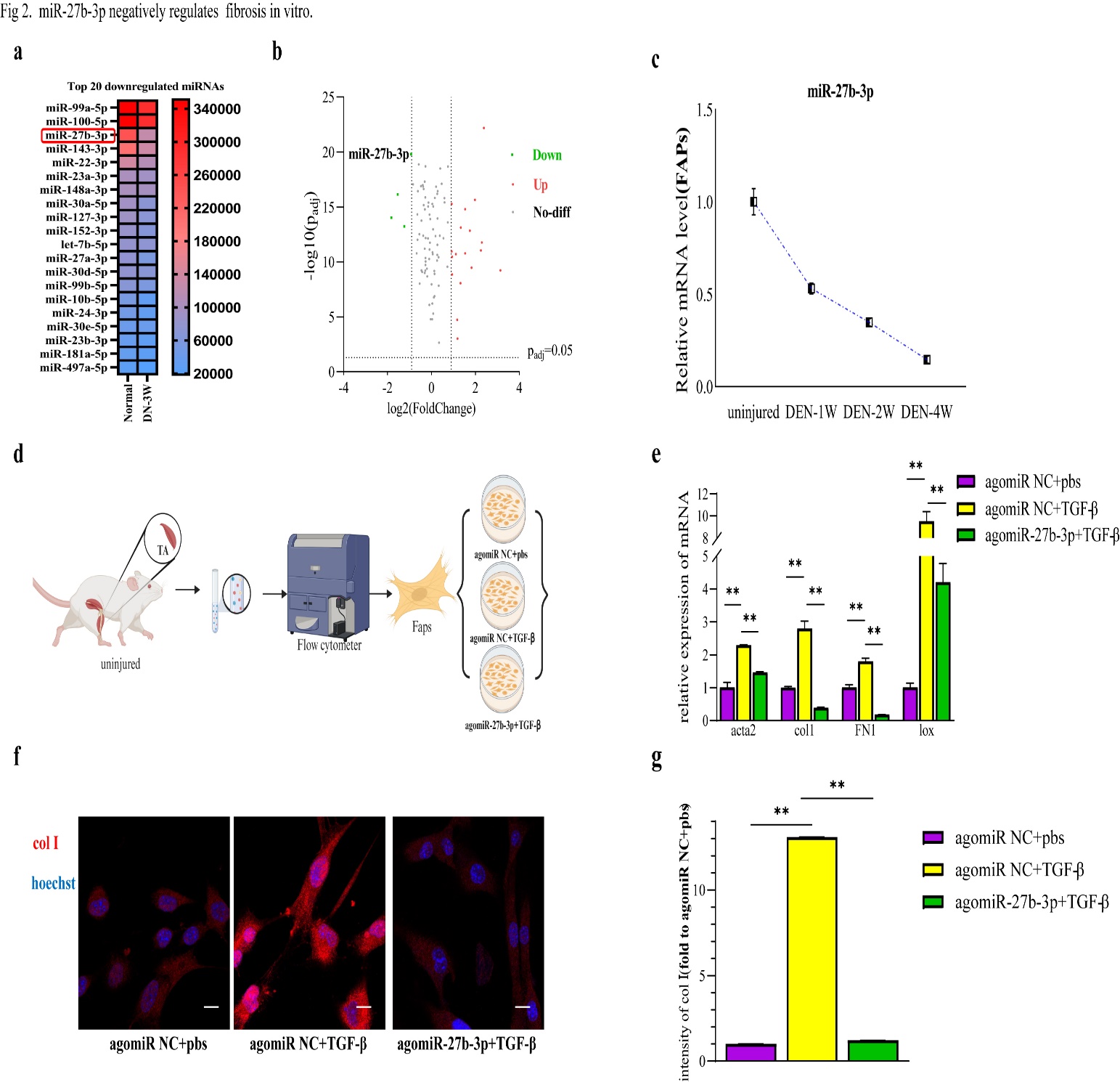


**FIGURE2** miR-27b-3p efficiently decreases fibrogenic differentiation of FAPs in vitro. **a, b,** heat map and volcano plots showed the miRNAs with different levels between FAPs isolated from DEN-3W and uninjured mice. **c,** relative expression of miR-27b-3p in purified FAPs isolated from uninjured muscles and denervated muscles. **d,** a schematic showing the experiment in vitro. **e,** the level of acta2, FN1, lox and col1 in FAPs induced by TGF-β and combined transfection with agomiR-27b-3p or agomiR NC were analyzed by qRT-PCR.  **f****, g,** Immunofluorescence of col1 and intensity of col 1. scale bar, 100um. All data are represented as mean ± SEM (n = 3). *P < .05, **P < .01.


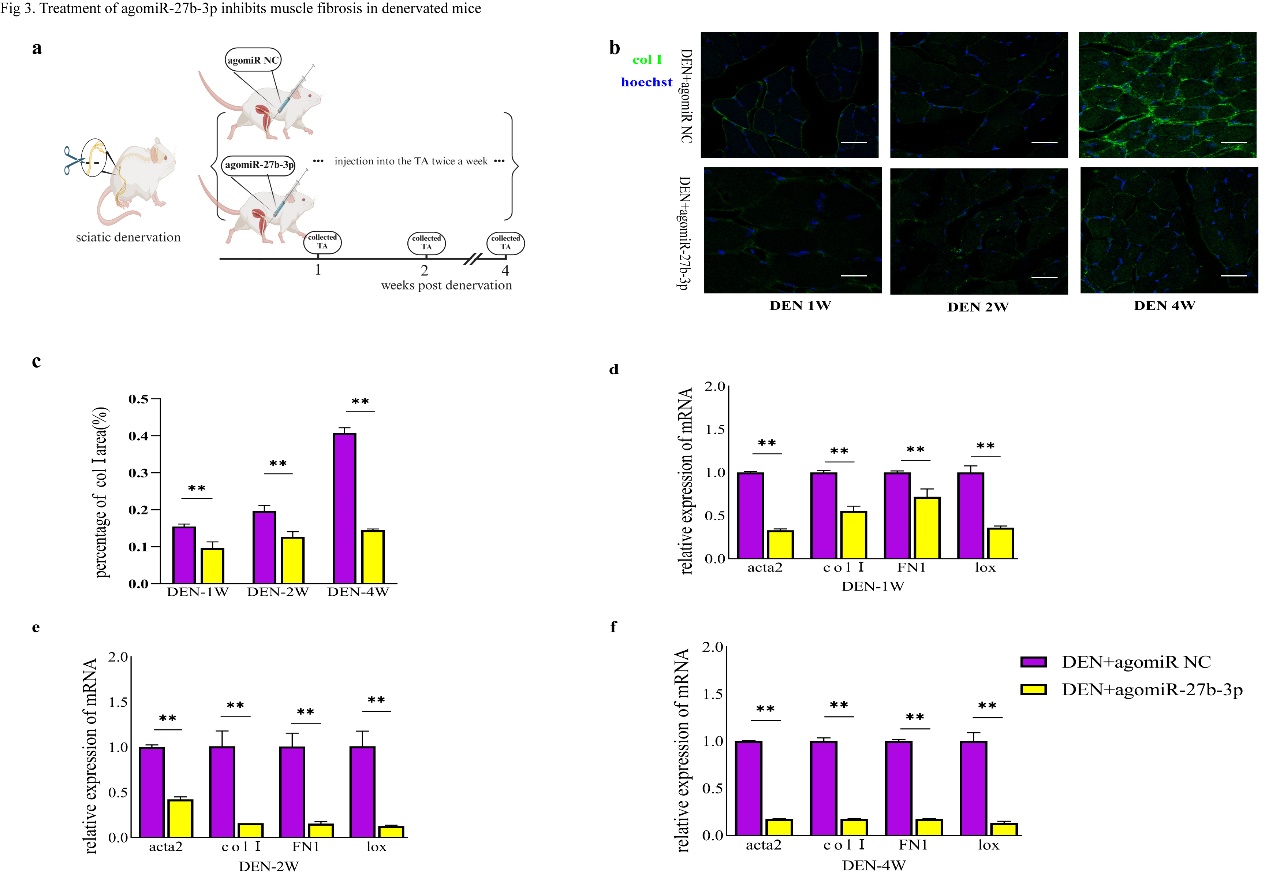


FIGURE3 agomiR-27b-3p suppresses muscle fibrosis in denervated mice. **a.** a schematic showing the experiment: injected agomiR NC or agomiR-27b-3p in denervated TA twice a week. **b, c,** Immunofluorescence for col1 of TA collected from denervated mice treatmented with agomiR NC or agomiR-27b-3p, and the percentage of area of col1, scale bar, 100um. **d, e, f,** relative expression of fibrogenic markers, acta2, FN1, lox and col1 in purified FAPs isolated from TA treatmented with agomiR NC or agomiR-27b-3p at different time points were examined by qRT-PCR. The legends of the three figures are consistent and are shown in the figure f. All data are represented as mean ± SEM (n = 3). *P < .05, **P < .01.


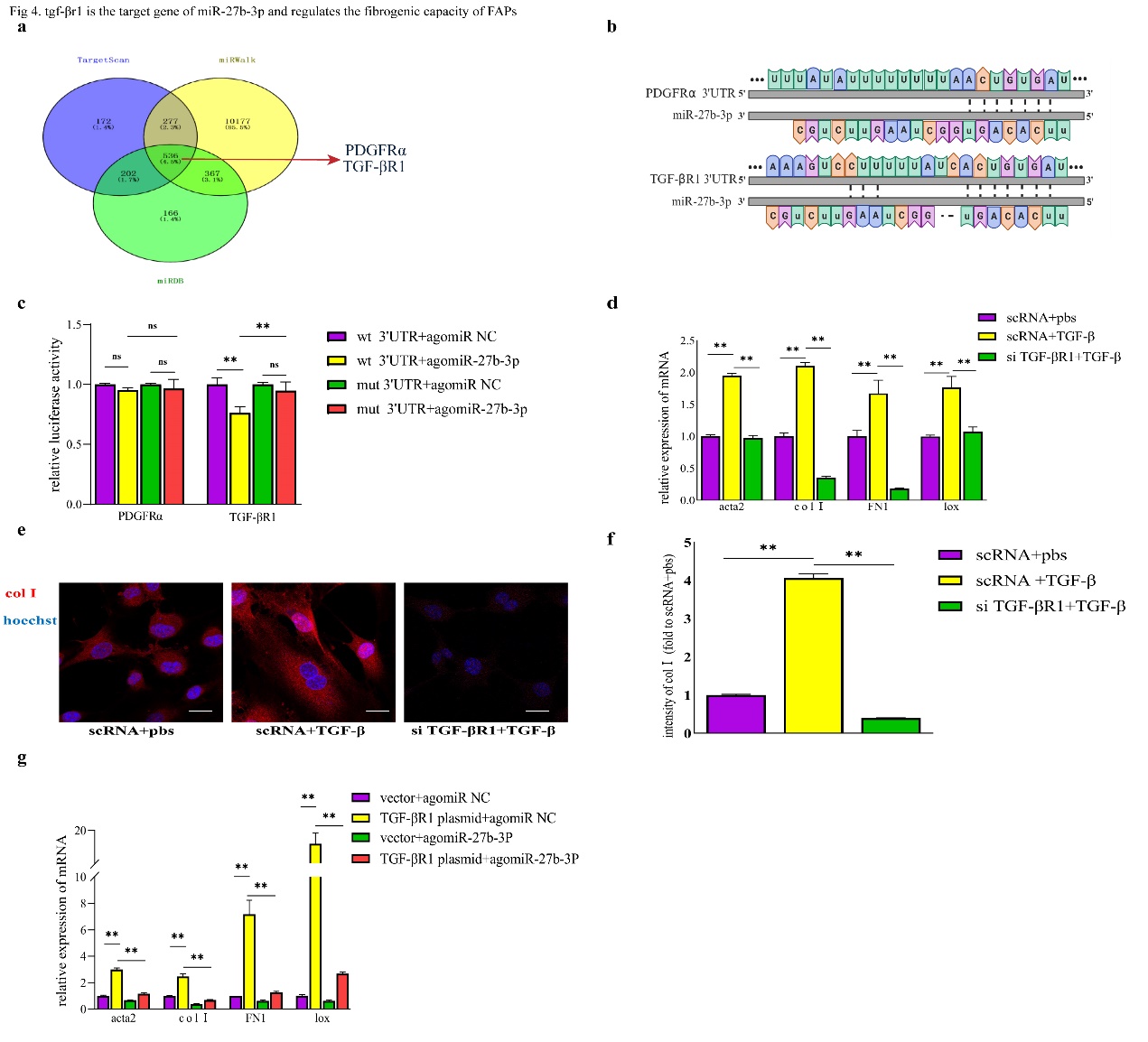


**FIGURE4.** TGF-βR1 is the target gene of miR-27b-3p and regulates the fibrogenic capacity of FAPs. **a,** Bioinformatics analysis was used to predict the potential target genes of miR-27b-3p. **b****,** A schematic showing predicted binding site of miR-27b-3p and the 3’UTR of candidate target genes’ mRNAs. **c.** The luciferase activity in 293T cells was tested using the Dual-Luciferase Reporter System. **d,** the expressions of fibrogenic genes in TGF-β-induced FAPs transfected with scRNA or si TGF-βR1. **e, f,** Immunofluorescence for col1 and average intensity of col1 in TGF-β-induced FAPs transfection with scRNA or si TGF-βR1, scale bar,100um. **g,** the mRNA expressions of acta2, FN1, lox and col1 in freshly purified Faps combined treatment with or without si TGF-βR1 plus plasmid with high expression of TGF-βR1 were examined by qRT-PCR. All data are represented as mean ± SEM (n = 3). *P < .05, **P < .01.


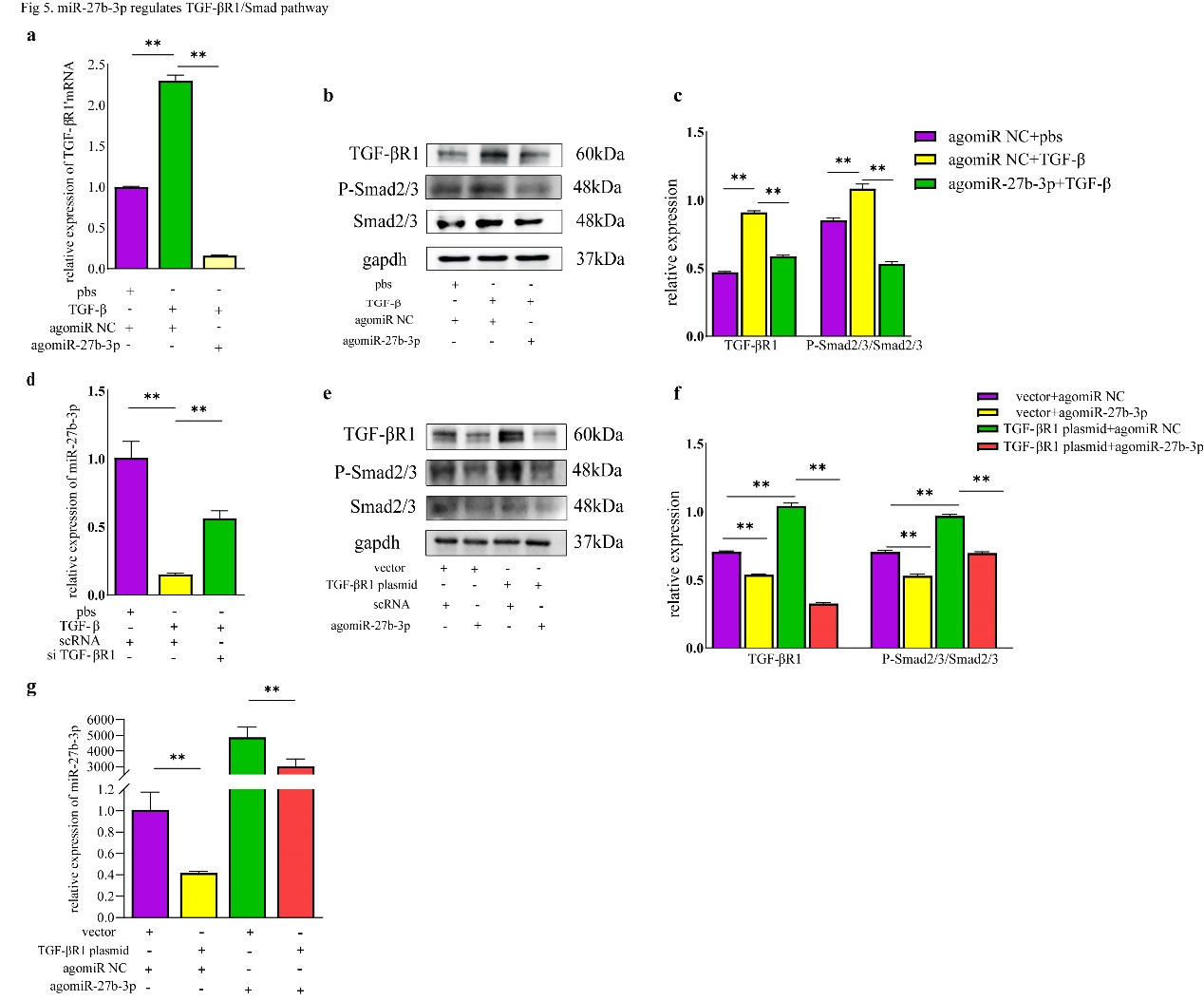


**FIGURE5** miR-27b-3p regulates fibrogenic capacity of FAPs through TGF-βR1/Smad pathway. **a,** mRNA expressions of TGF-βR1 in FAPs induced by TGF-β and followed by transfection with agomiR-27b-3p or agomiR NC. **b, c,** the protein expression of TGF-βR1, P-Smad2/3 and Smad2/3 in FAPs induced by TGF-β and transfected with agomir-27b-3p or agomiR NC were assayed by WB, and the relative intensity of ratio of TGF-βR1 and P-smad2/3/Smad2/3 were quantified. **d,** relative expression of miR-27b-3p in FAPs transfected with sc RNA or si TGF-βR1. **e, f,** the protein expression of TGF-βR1, P-Smad2/3 and smad2/3 in FAPs combined treatment with agomiR NC or agomiR-27b-3p plus plasmid with overexpression of TGF-βR1 were tested by WB, and the relative intensity of TGF-βR1 and the ratio of P-smad2/3/Smad2/3 were quantified. **g,** the abudance of miR-27b-3p in FAPs combined treatment with agomiR NC or agomiR-27b-3p plus plasmid with overexpression of TGF-βR1. All data are represented as mean ± SEM (n = 3). *P < .05, **P < .01.


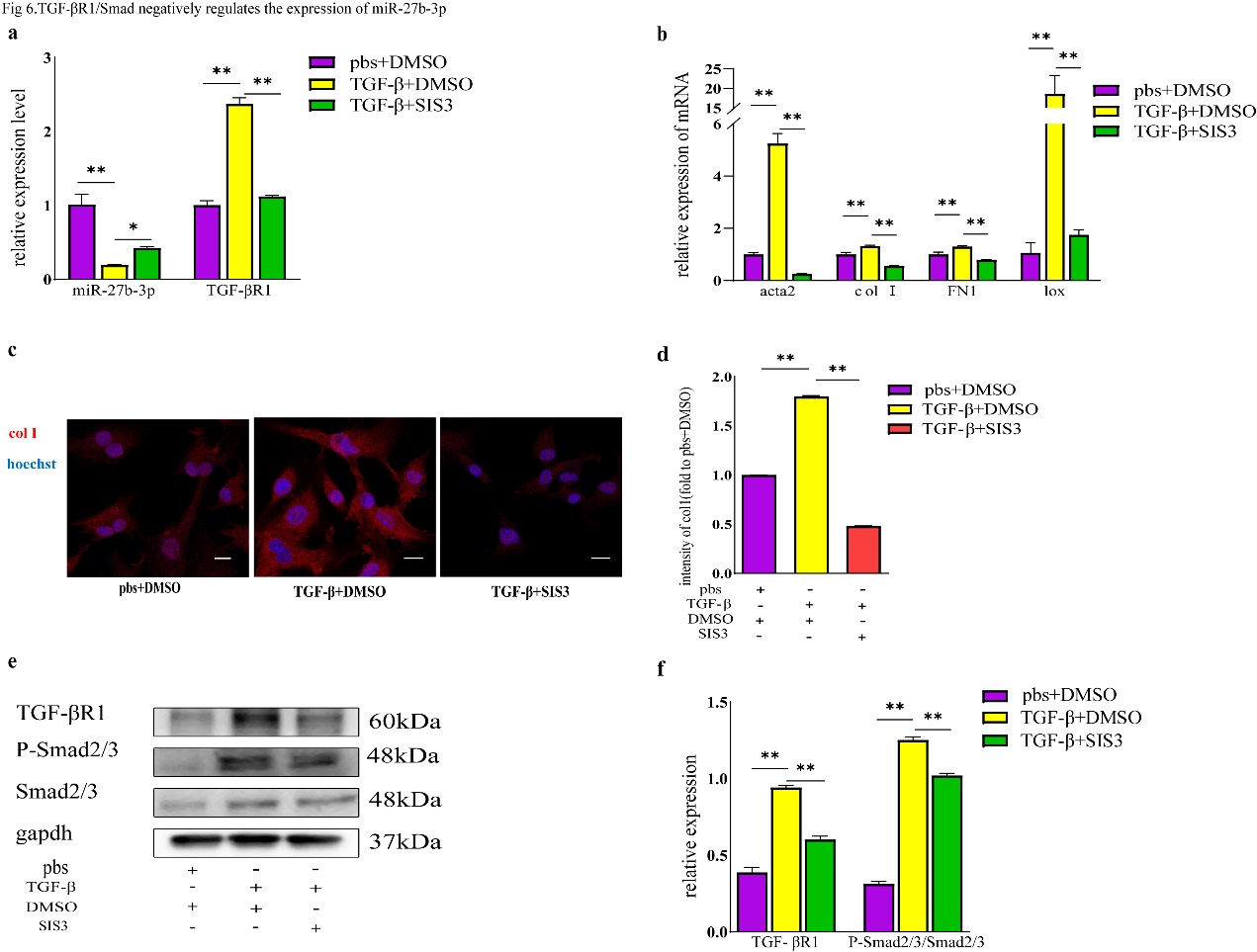


**FIGURE6** TGF-βR1/Smad negatively regulates the abundance of miR-27b-3p. **a,** **b,** the mRNA expressions of miR-27b-3p, TGF-βR1, acta2, FN1, lox and col1 in FAPs induced by TGF-β and followed by treatment with or without SIS3 in vitro were examined by qRT-PCR. **c, d,** Immunofluorescence for col1 and average intensity of col1 showed the fibrosis level in FAPs induced by TGF-β and followed by treatment with or without SIS3, scale bar, 100um. **e, f,** the protein expression of TGF-βR1, P-Smad2/3 and Smad2/3 in FAPs induced by TGF-β and followed by treatment with or without SIS3, and the relative intensity of TGF-βR1 and the ratio of P-Smad2/3/Smad2/3 were quantified. All data are represented as mean ± SEM (n = 3). *P < .05, **P < .01.


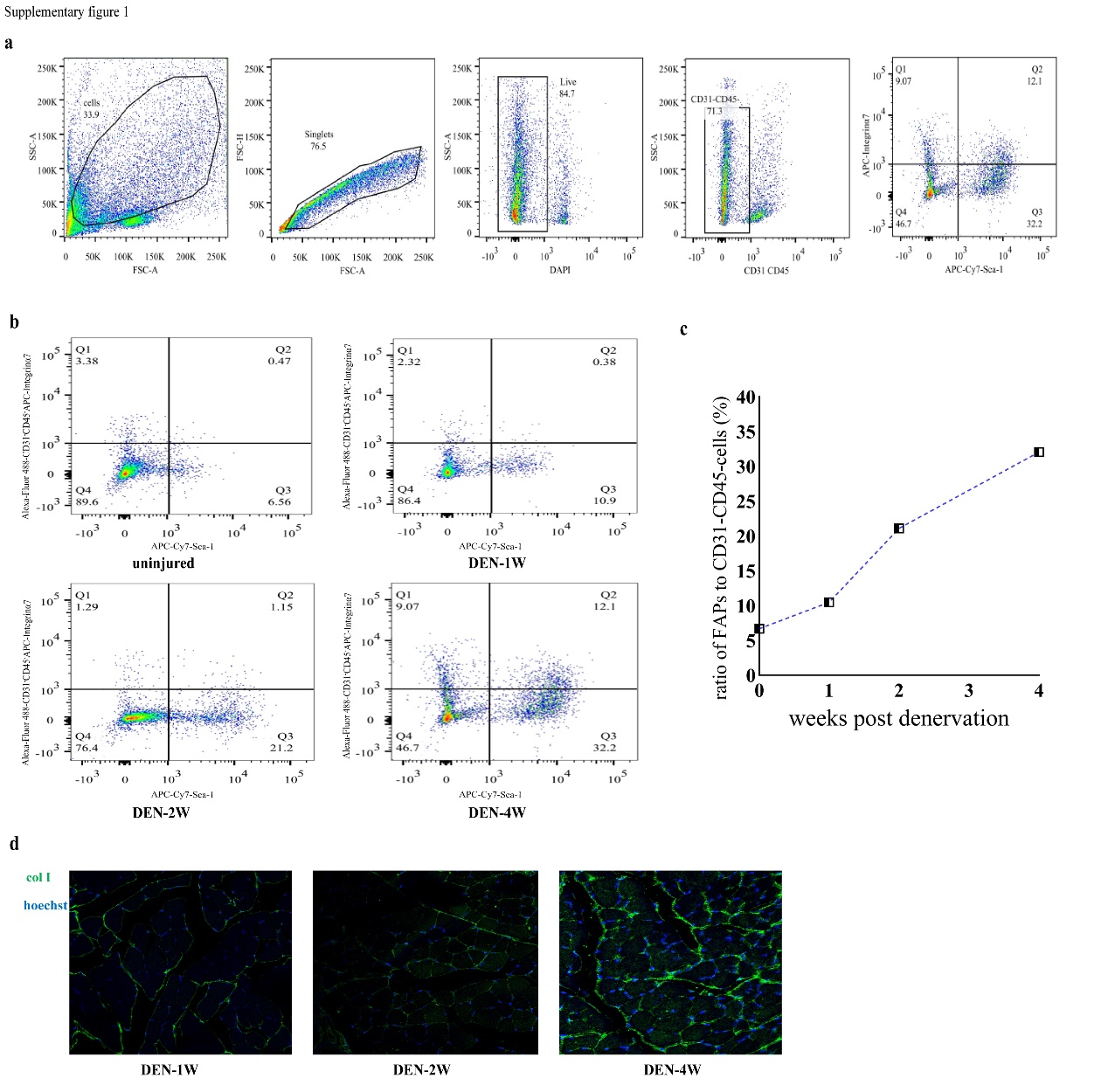


**Supplementary figure 1: a,** the gating strategy for isolating FAPs. **b, c,** the proportion of FAPs to CD31-CD45- cells in uninjured mice and mice with denervation. **d,** immunofluorescence for col 1 of TA collected from denervated muscles and the percentage of area of col1, scale bar,200um**.** All data are represented as mean ± SEM (n = 3). *P < .05, **P < .01.


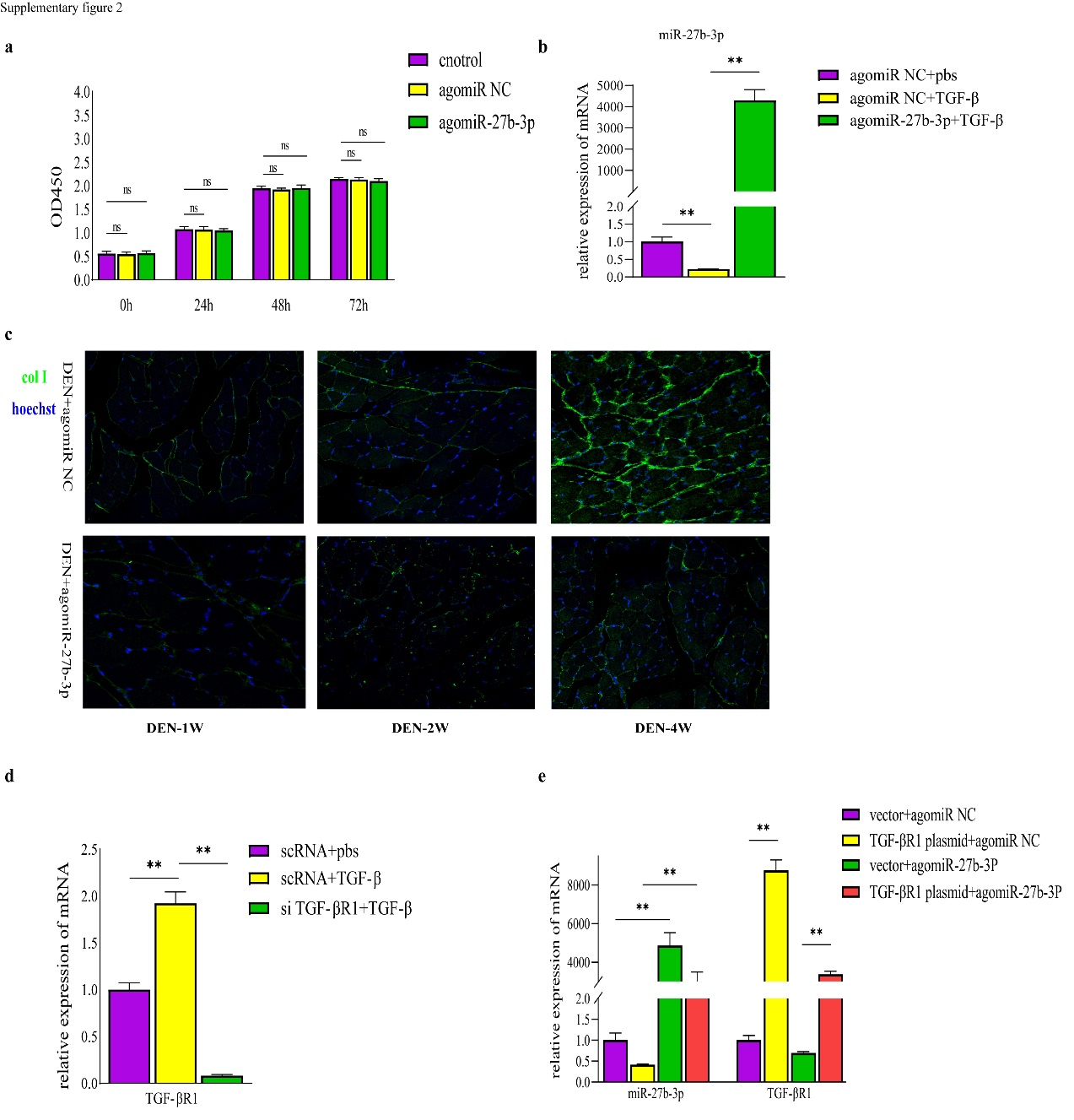


**Supplementary figure 2: a,** values of OD450 in CCK-8 assay. **b,** mRNA expression of miR-27b-3p after transfection with agomiR-27b-3p was tested by qRT-PCR to illustrate the transfection efficiency of agomiR-27b-3p. **c,** immunofluorescence for col1 of TA collected from denervated mice treatmented with agomiR NC or agomiR-27b-3p, scale bar, 200um. **d,** mRNA expression of TGF-βR1 after transfection with si TGF-βR1 was tested by qRT-PCR to illustrate the transfection efficiency of si TGF-βR1. **e,** mRNA expression of miR-27b-3p and TGF-βR1 after transfection with agomiR NC or agomiR-27b-3p plus TGF-βR1 plasmid to illustrate the transfection efficiency of TGF-βR1 plasmid. All data are represented as mean ± SEM (n = 3). *P < .05, **P < .01.
